# Physical, Mechanical, and Water Vapor Barrier Properties of Starch/Cellulose Nanofiber/Thymol Bionanocomposite Films

**DOI:** 10.3390/polym13234060

**Published:** 2021-11-23

**Authors:** Siti Hajar Othman, Bilguisse Mamadou Wane, Norhazirah Nordin, Noor Zafira Noor Hasnan, Rosnita A. Talib, Joko Nugroho Wahyu Karyadi

**Affiliations:** 1 Department of Process and Food Engineering, Faculty of Engineering, Universiti Putra Malaysia, Serdang 43400, Selangor, Malaysia; bilbandel@gmail.com (B.M.W.); nohaf27@gmail.com (N.N.); noorzafira@upm.edu.my (N.Z.N.H.); rosnita@upm.edu.my (R.A.T.); 2 Institute of Advanced Technology, Universiti Putra Malaysia, Serdang 43400, Selangor, Malaysia; 3 Department of Agricultural Engineering, Faculty of Agricultural Technology, Universitas Gadjah Mada, Yogyakarta 55281, Indonesia; jknugroho@ugm.ac.id

**Keywords:** biopolymer, corn starch, film, cellulose nanofiber, nanocomposite, thymol

## Abstract

The application of starch films, such as food packaging materials, has been restricted due to poor mechanical and barrier properties. However, the addition of a reinforcing agent, cellulose nanofibers (CNF) and also thymol, into the films, may improve the properties of films. This work investigates the effects of incorporating different concentrations of thymol (3, 5, 7, and 10 wt.%) on physical, mechanical, water vapor barrier, and antibacterial properties of corn starch films, containing 1.5 wt.% CNF produced using the solvent casting method. The addition of thymol does not significantly affect the color and opacity of the films. It is found that the tensile strength and Young’s modulus of the films decreases from 10.6 to 6.3 MPa and from 436.9 to 209.8 MPa, respectively, and the elongation at break increased from 110.6% to 123.5% with the incorporation of 10 wt.% thymol into the films. Furthermore, the addition of thymol at higher concentrations (7 and 10 wt.%) improved the water vapor barrier of the films by approximately 60.0%, from 4.98 × 10^—9^ to 2.01 × 10^—9^ g/d.m.Pa. Starch/CNF/thymol bionanocomposite films are also found to exhibit antibacterial activity against *Escherichia coli*. In conclusion, the produced starch/CNF/thymol bionanocomposite films have the potential to be used as antibacterial food packaging materials.

## 1. Introduction

Petroleum-based plastics, such as polyethylene (PE), polypropylene (PP), and polyamide (PA), are often used for food packaging due to their good physicochemical and processing properties [1]. Unfortunately, this leads to a large percentage of global plastic pollution, as they are non-degradable and inconvenient to be recycled because they are usually contaminated with food. The environmental impact of this issue has raised huge concerns and leads to a growing interest in developing bio-based polymers or biopolymers as biodegradable films for food packaging.

Among the biopolymers, starch is a highly promising polysaccharide to formulate biodegradable films. Starch is abundant, biodegradable, economical, and exhibits a good film-forming property [2]. Starch is the main source of glucose in many types of plant organs, such as the seeds, tubers, roots, and fruits [3]. Starch is widely available in a few natural sources, such as corn, wheat, and potatoes. Among the various sources of starch, corn starch has the highest amylose content, which can vary from 22.4 to 67.8% [4]. Given that the film-forming properties of starch are dependant on the amylose content in the starch, films produced from corn starch are more rigid and strong [5].

However, for food packaging applications, starch-based films are limited by their poor mechanical and barrier properties [6]. To improve the granular structure and the functional properties of starch films, nanosized fillers and antimicrobial and/or antioxidants agents can be incorporated into the starch films [3]. The formulation of starch films mixed with nano-sized fillers, such as cellulose nanofibers (CNF) to form bionanocomposite, has been proven to improve the mechanical, thermal, and barrier properties of the films. The good adhesion between CNF and starch matrix in starch-based films, which attributes to the similarity of the chemical structure of the two components, facilitates the bonding and improvement of the mentioned properties [7].

Apart from that, fresh food products can be easily spoiled by spoilage microorganisms during processing, distribution, and market exposure, thereby causing deterioration and food waste. This phenomenon has a significant negative economic impact on the food industry and is also detrimental to consumers’ safety. Since microbial contamination mainly occurs at the surface of many refrigerated food products [8], packaging materials can therefore be a part of the solution to control such contamination. The addition of antimicrobial agents into food packaging films can inhibit and kill microbes that spoil food, thus extending the shelf-life of the food product. Among the antimicrobial agents, thymol (C_10_H_14_O) is a promising antimicrobial agent for antimicrobial packaging due to the broad spectrum of antimicrobial activity against foodborne pathogens, such as *Salmonella typhimurium*, *Listeria monocytogenes*, *Escherichia coli* (*E. coli*), *Staphylococcus aureus*, and *Bacillus subtilis* [9]. Thymol is a phenolic compound that can be extracted from the aromatic plant thyme (*Thymus vulgaris*) and is known as an excellent antimicrobial agent.

However, the addition of different concentrations of thymol may affect the physical, mechanical, barrier, and antimicrobial properties of the resulting films. Therefore, this work aims to investigate the physical, mechanical, water vapor barrier, and antibacterial properties of the starch/CNF bionanocomposite films incorporated with different concentrations of thymol (starch/CNF/thymol bionanocomposite films). The antibacterial activity of the films was investigated against *E. coli* through the disc diffusion method. The real application of the films was demonstrated on fresh meat samples. Fresh meat was chosen, as it is susceptible to bacterial growth, particularly by *E. coli*. The most commonly associated serotype is *E. coli* O157:H7, which is naturally found in the intestines of animals, such as cattle, sheep, and goats [10]. Furthermore, the meat surface can be contaminated with *E. coli* during the slaughtering process and subsequent handling [11]. Thymol is known to be effective against a wide spectrum of bacteria, specifically *E. coli*, which is the most prevalent meat spoilage agent. [12].

To the best of our knowledge, no work has been performed to produce corn starch/CNF bionanocomposite films incorporated with thymol at 3, 5, 7, and 10 wt.% concentrations. Only one study by Othman et al. [7] has investigated the effects of CNF and different concentrations of thymol on the mechanical, thermal, and barrier properties of corn starch films. Nonetheless, the concentrations of thymol that were used were different from this work. Apart from that, the investigation on the antibacterial activity of the films against *E. coli*, as well as the demonstration on the applicability of the films as potential active food packaging materials on fresh meat samples, has not yet been conducted. Due to its promising potential as an active food packaging material, it is important to determine the active properties of the films, specifically the antibacterial activity of the films, and demonstrate the real application of the films on food.

## 2. Materials and Methods

### 2.1. Materials

The materials used in this study were food-grade corn starch (33% amylose, 67% amylopectin) purchased from R&M Marketing, Semenyih, Malaysia, CNF suspension (concentration: ~2% *w*/*v*, size: diameter <80 nm, length 10–100 μm, structure: entangled fibrous, pH: 7, crystallinity: 40%) purchased from the Institute of Tropical Forestry and Forest Product, Universiti Putra Malaysia (UPM), Serdang, Malaysia, thymol crystal, glycerol, calcium chloride (CaCl_2_), sodium bromide (NaBr), paraffin wax, beeswax, and ethanol purchased from R&M Marketing, Semenyih, Malaysia, and Tween 80 (polyoxyethylene-20-sorbitan monooleate) purchased from Sigma–Aldrich, St. Louis, MO, USA.

*E. coli* bacteria (ATCC 25922, clinical isolates) were obtained from the Microbiology Laboratory, Faculty of Food Science and Technology, UPM, Serdang, Malaysia. Nutrien agar and Mueller–Hinton agar (MHA) were purchased from Merck, Darmstadt, Germany. Peptone water was purchased from Friendemann Schmidt, Washington, WA, USA, while the meat slices (beef) were bought from HeroMart, Sri Serdang, Malaysia.

### 2.2. Preparation of the Films

**Figure 1:**
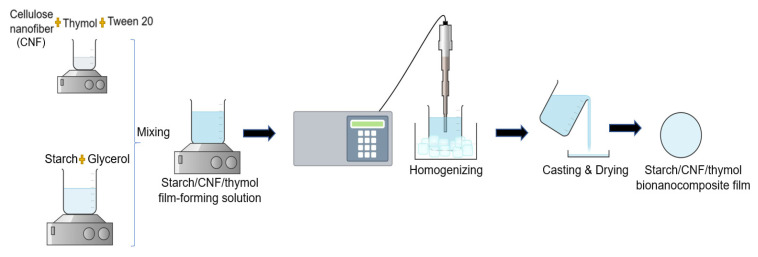
Schematic diagram of the film preparation procedures.

Starch/CNF/thymol bionanocomposite films were prepared using the solvent casting method, according to the work of Othman et al. [7], as shown in Figure 1, but with some modifications (different concentrations of thymol, dissolved thymol in a higher amount of distilled water and surfactant, and a longer stirring time) to produce a more homogenous starch/CNF/thymol film-forming solution, thus improving the properties of the films. For the preparation of the film-forming solution, an amount of 8 g corn starch and 2 g glycerol (25 wt.% of starch) were dissolved in 160 mL of distilled water. The solution was heated for 30 min and stirred constantly using a hotplate magnetic stirrer (DAIHAN, Jakarta, Indonesia) at 700 rpm until it gelatinized at 90 °C. Then, the film-forming solution was left to cool down to 50 °C at ambient temperature. In the meantime, CNF/thymol emulsion was prepared by adding various amounts of thymol (3, 5, 7, and 10 wt.% of starch) into 40 mL of distilled water containing 1.5 wt.% of CNF (6 mL) at a temperature of 50 °C. The 1.5 wt.% concentration of CNF was chosen based on the previous work by Othman et al. [7], whereby this amount of CNF was recorded to produce starch films with the most optimal mechanical and barrier properties. A higher amount of CNF was found to reduce the mechanical strength and water vapor permeability of the films. Thymol is known to be volatile, therefore the temperature of 50 °C was fixed to minimize the loss of thymol via volatilization during the preparation.

Subsequently, a fixed amount of Tween 20 (20 wt.% of thymol) was added as a surfactant and the emulsion was continuously stirred for 15 min using a digital hotplate stirrer. The CNF/thymol emulsion was added to the gelatinized starch film-forming solution in droplets using pipettes, after the film-forming solution was cooled down. The solution then underwent ultrasonication (QSonica, 500 W, 20 kHz) for 10 min at 50% amplitude to produce a homogenous solution. The solution was then casted in a 140 mm Petri dish (35 mL each) and dried in an air-conditioned room (21 °C) for 48 h. All the films were then placed in a ventilated oven (Memmert Universal Oven UN110, Schwabach, Germany) at 45 °C for 15 min to dry. The dried films were peeled off from the casting plate and conditioned in a dry cabinet (WEIFO, Selangor, Malaysia) which was fixed at 25 °C and 55% relative humidity (RH) for 48 h before proceeding with characterization. Starch/CNF bionanocomposite films without the addition of thymol were also prepared as the control using the explained method.

### 2.3. Characterization of the Films

#### 2.3.1. Physical Properties

The physical properties of the films were determined in terms of thickness, color, opacity, and appearance. The thickness of the films was measured using a digital micrometer (Mitutoyo, Kawasaki, Japan). The color of the films was determined using a color spectrophotometer (Hunterlab, Ultrascan Pro, VA, USA). The CIELAB color coordinates (L*, a*, and b*) with a scale of L* = 0 (black) to L* = 100 (white), −a* (greenness) to +a* (redness) and −b* (blueness) to +b* (yellowness) were used. The total color difference (ΔE) was calculated using the following equation:

ΔE = [(L* − L)^2^ + (a* − a)^2^ + (b* − b)^2^]^1/2^ (1)

The light transmittance of the films was recorded using the color spectrophotometer from a wavelength of 200 nm to a wavelength of 700 nm. The transmittance value measured at 600 nm was used to calculate the opacity using the following equation:

Opacity = A600/L(2)

where A600 is the value of absorbance at wavelength 600 nm and L is the film thickness (mm). Meanwhile, the appearance of the films was observed by taking the images of the films on the printed letter ‘P’.

#### 2.3.2. Mechanical Properties

The films were first cut into rectangular strips (100 mm × 15 mm). Texture analyzer (TA.XT2 Stable Micro Systems, Surrey, UK) was used to determine the tensile strength (TS), elongation at break (EAB), and Young’s modulus (YM) according to the American Society for Testing and Materials (ASTM) D882 [13]. The films were stretched at a speed of 20 mm/min. A microcomputer was used to record the stress–strain curve.

#### 2.3.3. Water Vapor Barrier

The water vapor barrier was determined by establishing the water vapor permeability (WVP) of the films using a dry cup method according to ASTM E96 [14]. First, the films were cut into circular shapes using a cutter with a diameter of 7 cm and a transmission area of 28 cm^2^. A test dish inside the permeability cup was filled with 10 g of CaCl_2_ to ensure a 0% RH below the film. The film was placed on top of the permeability cup and closed with a ring cover. The cup and the ring were sealed with a mixture of melted paraffin wax and beeswax (8:2). The cup was then placed in a desiccator containing a magnesium nitrate saturated solution to provide the RH of 54% at 25 °C. Inside the desiccator, the temperature and relative humidity were monitored using a digital temperature humidity meter (Proskit NT-312, Techno Tools & Equipment Sdn Bhd, Selangor, Malaysia). The weight of the permeability cup was measured every 24 h for 10 days and the weight versus time graph was plotted. The water vapor permeability rate (WVTR) and the WVP of the films were calculated using Equations (3) and (4), respectively:

WVTR = (g/t)/A(3)

WVP = WVTR × L/ΔP(4)

where g/t is the slope of the straight line from the graph of weight changes versus time (g/day), A is the transmission area of the film (m^2^), L is the average thickness of the film (m), and ΔP is the vapor pressure difference between the inside and outside of the cup (RH 54%–RH 0%), ΔP = 2.29 × 10^6^ Pa.

### 2.4. Antibacterial Activity of the Films

The antibacterial activity of the films against *E. coli* was investigated in vitro via a disc diffusion assay. *E. coli* was cultivated on nutrient agar and incubated for 24 h at 37 °C in an incubator (Thermo Scientific Heraeus Function Line Incubator, Shanghai, China). Then, a suspension of the *E. coli* bacteria was prepared in peptone water, such that the concentration of the bacteria was approximately 10^7^–10^8^ CFU/mL. An amount of 0.1 mL of the bacteria suspension was spread over the prepared MHA surface in a Petri dish. Film samples were cut into circles (6 mm diameter) using a cutter and sterilized for 30 min using UV light of the laminar flow (Esco Global, Changi South Lane, Singapore). Under the laminar flow, all the discs were carefully placed into the Petri dish that was previously inoculated. The plates were incubated for 24 h at 37 °C. The presence of a clear halo inhibition zone around each of the film discs indicated the antibacterial activity of the films. After the incubation, the diameter of the halo inhibition zone was measured.

To assess the applicability of the films as potential active food packaging materials, the films were applied on fresh meat samples, whereby each film was placed in direct contact with the meat samples. The method for in vivo analysis followed the previous studies by Guo et al. [15]. The meat and the films were first cut aseptically into 3 cm × 3 cm and 5 cm × 5 cm, respectively. The meat samples were then sprayed with 75% *v/v* ethanol and inoculated with 0.1 mL of *E. coli* suspension. The inoculum was spread evenly on the upper surface of the meat using a sterile spreader. All the steps were conducted under a laminar flow to prevent external contamination. Afterward, the meat sample was placed perfectly at the center of the film. As a control, the inoculated meat samples in the absence of the films were also prepared.

The samples were each put in a Petri dish and kept in a chiller (13 °C) to stimulate mild temperature abuse [16]. Then, on different reading days (1, 3, and 7), the images of the meat samples were captured. Next, each meat sample was placed in a stomacher bag and macerated with 400 mL of 0.1% *w/v* peptone water for 2 min. The meat samples were filtered and serially diluted in peptone water. Then, 0.1 mL of the final dilutions were plated onto MHA and incubated at 37 °C for 24 h. Finally, the number of colonies formed was counted, taking into account the dilution factor.

### 2.5. Statistical Analysis

The statistical analysis of the experimental results was conducted using Minitab software (Minitab Inc., Version 16, State College, PA, USA). The experimental results were presented as a mean value ± standard deviation of triplicate measurement (n = 3). The analysis of variance (ANOVA) was utilized to evaluate the significant difference between the means. The Tukey test was used to compare the mean values, whereby the differences between means were considered to be significant if *p* < 0.05.

## 3. Results and Discussion

### 3.1. Physical Properties

Table 1 shows the thickness, color parameters, and opacity of the films. From Table 1, it can be observed that the thickness of the starch/CNF bionanocomposite films increased slightly with the addition of thymol and the thickness also increased slightly with the increase in the concentration of thymol, even though the volume of the film-forming solution was set to cast at 35 mL. This was due to the plasticizing effect of thymol, whereby the intermolecular biopolymer chains started to restructure, resulting in voids and leading to thicker films [5]. This finding correlates with the work of Li et al. [17] who found that the thickness of gelatin films increased with the addition of thymol nanoemulsions. The films also showed similar behavior to that described by LozanoNavarro et al. [18], whereby adding the natural extract from oregano to chitosan–starch films resulted in an increasing trend of the thickness of the films. This was due to the presence of compounds, such as polysaccharides, carboxylic acids, and antioxidants in the extract, which resulted in a more complex matrix. Hence, in this study, the presence of thymol, which is a natural extract, increased the thickness of the films with the increase in the concentration of thymol.

From Table 1, it can be seen that there were only slight differences in the L*, a*, and b* values for the starch/CNF and starch/CNF/thymol bionanocomposite films that have been incorporated with various concentrations of thymol, indicating that all films were almost similar in color, and are thus suitable for food packaging applications. It was found that the color difference (ΔE), increased slightly with the increase in thymol concentration, due to the white crystalline color of thymol. This result was consistent with the findings of Othman et al. [7]. Hosseini et al. [19] also found that the ΔE values of chitosan-based films incorporated with thyme essential oil increased with the incorporation of the essential oil.

On the other hand, a low opacity value indicates high transparency of the film and vice versa. Transparency of food packaging material is a crucial factor because it can help suppliers to keep track of the product quality through distribution and storage, as well as help appeal to consumers [20]. According to Garavand et al. [21],the film transparency for food packaging varies depending on the formulation of the film and it is preferred to have a high film transparency. It was found that the opacity of the films increased slightly with the increase in the concentration of the thymol, indicating that the transparency of the films became slightly reduced. These findings were consistent with the work of Zhong et al. [22] and Othman et al. [7], and attributed to the presence of phenolic compounds in the thymol that form an emulsion. Nordin et al. [4] stated that thymol is an oil compound that would produce an emulsion when incorporated into the film solution, thus influencing the opacity of the film.

**Figure 2:**
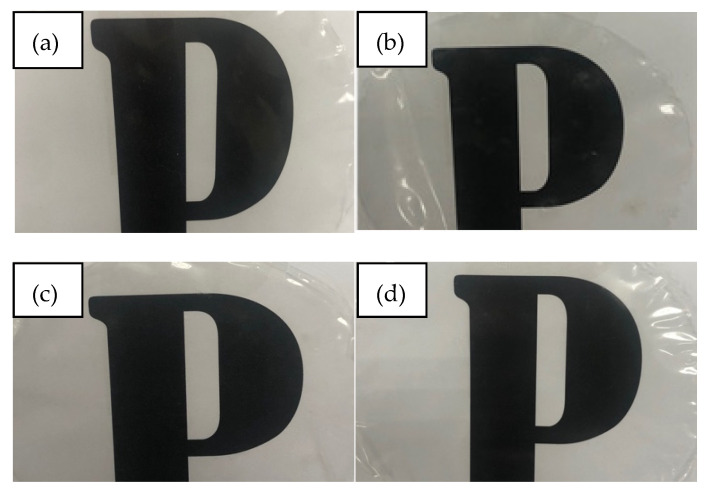
Physical appearance of starch/CNF bionanocomposite films incorporated with (**a**) 0, (**b**) 3, (**c**) 5, and (**d**) 10 wt.% of thymol.

Figure 2 shows the physical appearance of the films. No major difference was observed in terms of the physical appearance of the films, due to the very slight decrement in the color difference and opacity of the films. This finding indicated that the films still preserved good color properties and high transparency, and that they are suitable to be used for food packaging applications. It is worth noting that in comparison with the work of Othman et al. [7], in this research thymol did not cause any significant change in the appearance and the transparency of the starch/CNF/thymol films, because several modifications were made during the preparation of the films and particularly during the preparation of the CNF/thymol emulsion, whereby the thymol was dissolved in a higher amount of distilled water and surfactant. In addition, the stirring period was also extended. These modifications may have contributed to a more homogenous starch/CNF/thymol film-forming solution and thus improved the films in terms of appearance and transparency.

### 3.2. Mechanical Properties

**Figure 3:**
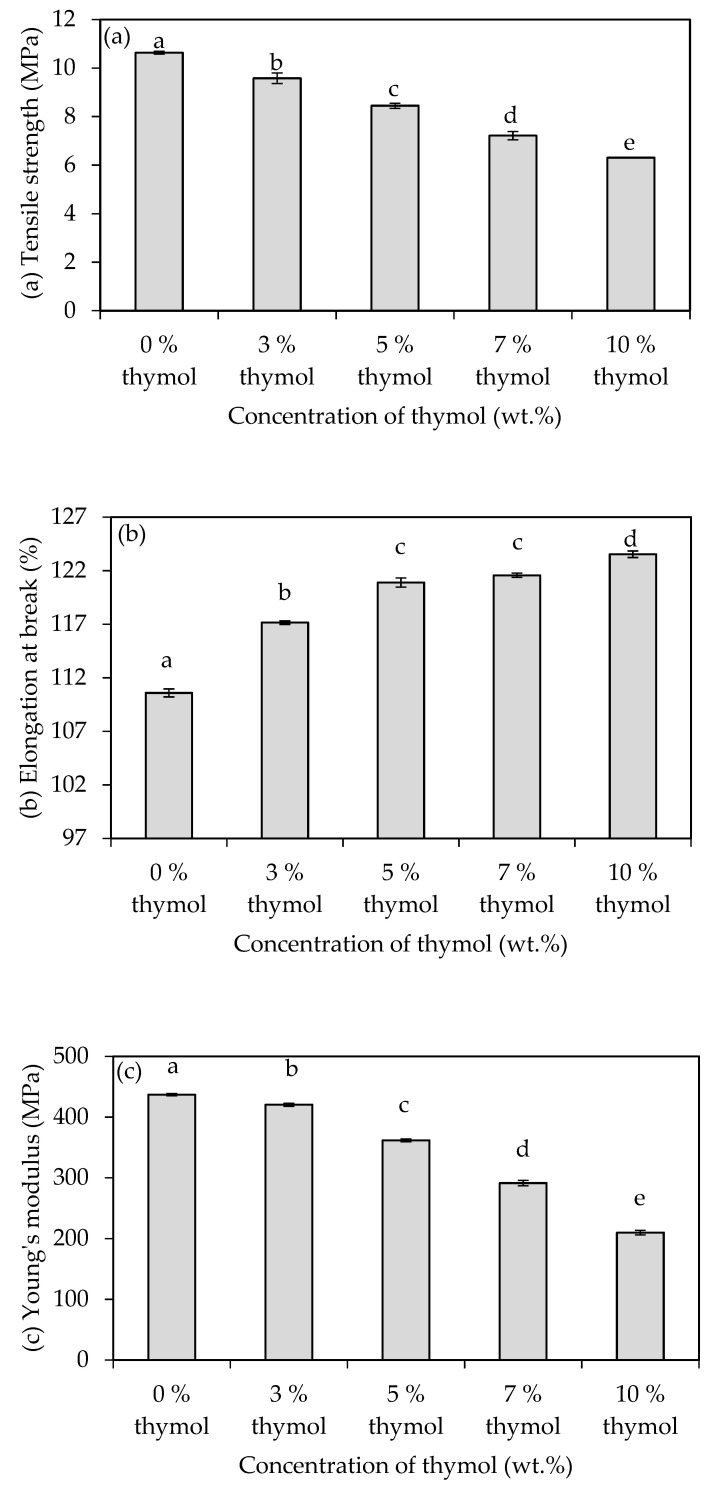
(**a**) Tensile strength, (**b**) elongation at break, and (**c**) Young’s modulus of starch/CNF bionanocomposite films incorporated with different concentrations of thymol. Different letters in the same graph indicate a statistically significant difference (*p* < 0.05).

Figure 3a shows the effects of incorporating different concentrations of thymol on the TS of the starch/CNF bionanocomposite films. The TS of the films decreased with the addition of thymol and the decrement became more pronounced at higher thymol concentrations. The TS of starch/CNF bionanocomposite films decreased by 40.7% when 10 wt.% of thymol is added, whereby the TS values decreased from 10.6 to 6.3 MPa. The resulting films containing 3 to 10 wt.% of thymol exhibited the TS values that were comparable with that of a common food plastic material, particularly low-density polyethylene (LDPE), whereby the range of TS values for LDPE is between 8.3 to 31.4 MPa [23]. This proves that the developed films have the potential to be used as food packaging material. Thymol is a hydrophobic agent that can penetrate polymer chains and reduce the intermolecular forces. This leads to the decrease in the rigidity of the films and consequently, the decrease in the TS values.

The same trend of findings was obtained by Othman et al. [7] and was attributed to the complex interaction formed between the lipids from the thymol and starch biopolymers, which could have reduced the cohesion of the starch network forces. Nonetheless, the tensile strength of the starch/CNF/thymol films found in this study was higher compared with the work of Othman et al. [7]. This observation may be attributed to the different formulations and process used to produce the films. The homogeneity of the film components in the matrix of the films enables better distribution of force exerted on the films, leading to better tensile strength. Other works, including Kavoosi et al. [24], found the same trend of results when they added thymol into gelatin films; they explained that the result was due to the plasticization effect of thymol making the films more soft. Furthermore, it was found that when thymol was incorporated into polybutylene succinate (PBS)-based films, the tensile strength was reduced by 10% to 40%, depending on the thymol content [25].

Figure 3b shows the effects of adding different concentrations of thymol into the starch/CNF bionanocomposite films on the EAB of the films. It can be seen that the EAB values increased with the addition of thymol and there was an increasing trend in EAB with the increase in concentrations of the thymol. The trend of the EAB values was reciprocal with the trend of the TS values, as expected. The starch/CNF bionanocomposite films without the thymol addition exhibited the minimum EAB value of 110.6%, whereas the films incorporated with 10 wt.% of thymol exhibited the maximum EAB value of 123.5%, which was 11.7% higher. All the resulting films exhibited EAB values in the range of that of common food plastic materials, particularly polyethylene terephthalate (PET), high-density polyethylene (HDPE), LDPE, and polypropylene (PP), which ranges from 30% to 300%, 10% to 1200%, 100% to 650%, and 100% to 600%, respectively [23]. This proves that the developed films have the potential to be used as food packaging materials.

A similar trend of results was also reported by Arfat et al. [26], who produced fish skin gelatin films incorporated with basil leaf essential oil, and Ansorena et al. [27], who incorporated thyme oil into polyethylene wheat gluten films whereby the EAB increased with the increase in the oil concentrations. These results can be explained by the changes in the crystallinity and the increase in ductile properties of the polymer, caused by the incorporation of the thymol. Othman et al. [7] in their work revealed that the crystallinity of the starch films decreased with the increase in thymol, thus improving the EAB. Thymol, which acts as a plasticizer, hinders chain-to-chain interactions in the films’ matrix and provides flexible domains in the films [7,28].

Figure 3c shows the YM of the starch/CNF bionanocomposite films incorporated with different concentrations of thymol. It can be observed that the incorporation of thymol caused a decreasing trend in YM values of the films, whereby the YM of the starch/CNF bionanocomposite films decreased from 436.9 to 209.8 MPa with the addition of 10 wt.% thymol, indicating a reduction in stiffness of the films. This result is consistent with the findings reported by Ramos et al. [29], in which the addition of 8 wt.% thymol into PP films significantly decreased the YM from 851 to 677 MPa. The YM values of the starch/CNF/thymol bionanocomposite films incorporated with 7 and 10 wt.% thymol in this study were consistent with the range of YM values of LDPE material, ranging between 172 to 282 MPa [23].

The reduction in the YM values with the addition of thymol can be explained by the plasticizing effect of thymol that can lead to a less stiff and more easily deformed film. The addition of thymol to the polymer matrix causes a modification of the crystallinity and decreases the ductile properties, thus improving the flexibility of the films [7,30] and subsequently, decreasing the YM. It is worth noting that the YM values of starch/CNF/thymol films found in this study were higher compared to the work of Othman et al. [7], and attributed to the homogeneity of the film components in the films’ matrix, as discussed previously, which contributed to a better distribution of force exerting the films and hence led to higher YM values.

### 3.3. Water Vapor Barrier

**Figure 4:**
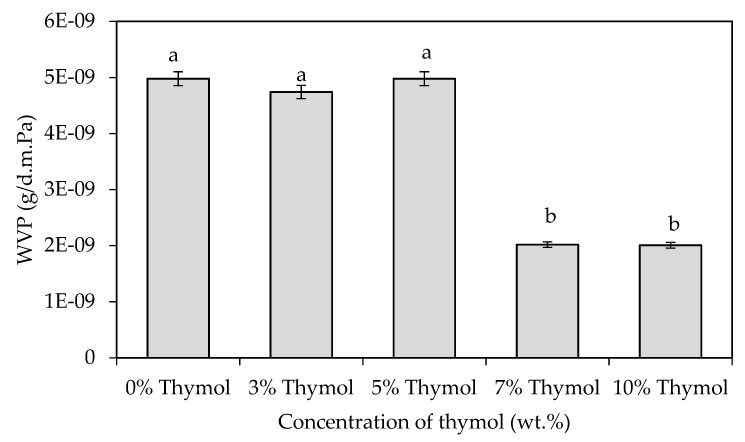
WVP of starch/CNF bionanocomposite films incorporated with different concentrations of thymol. Different letters in the same graph indicate a statistically significant difference (*p* < 0.05).

WVP indicates the water barrier efficiency of the films and is a crucial factor to monitor, as it can affect the quality of the food packaged within the films, especially during distribution and storage [31]. Figure 4 shows the WVP of the starch/CNF bionanocomposite films incorporated with different concentrations of thymol. It was found that at lower concentrations (<7 wt.%), thymol content was not enough to cause significant changes in the WVP. In this context, the dominance of the hydrophilic molecules, such as glycerol and starch, as well as a large number of free hydroxyl groups within the starch matrix, surpassed the presence of thymol in the film matrix, facilitating the diffusion of water molecules. This phenomenon corroborated the findings of Othman et al. [7], who found that the presence of thymol at low concentration (0.1% *w*/*v*) did not apparently alter the functional bondings between the film components, as proven by the FTIR analysis.

However, at higher concentrations (≥7 wt.%), thymol presence caused a decrease in the WVP of the films. The WVP of the films is dependent on the hydrophobic–hydrophilic balance of the components that form the films and the degree of cross-linking. It can be deduced that when thymol was introduced into the films at high concentrations with an appropriate dispersion, an efficient cross-linking occurred between the film components, and hydrophobic groups such as C=C and C=O contributed to the increase in hydrophobicity and water-resistance of the films. Many works explained that the incorporation of oils would lead to an increase in the hydrophobicity of the polymer-based films and thus, to a decrease in the WVP [32,33,34]. The work of Kalateh-Seifari et al. [33] reported a decreasing trend of WVP with an increasing concentration of nettle essential oil in corn starch/chitosan films. This result was attributed to the strong interactions between the starch/chitosan matrix and the phenolic compound via hydrogen and covalent bonds, which reduced the film’s tendency to water. They emphasized that water vapor permeation occurs through the hydrophilic parts of the film, and thus the permeability depends on the hydrophobic–hydrophilic ratio of the film components.

In the previous study by Othman et al. [7], the addition of 0.1% *w/v* thymol reduced the WVP of the starch/nanocellulose fiber bionanocomposite films. On the other hand, the addition of 0.3% and 0.5% *w/v* of thymol resulted in an increased WVP, due to the hindrance effect of thymol on polymer chain-to-chain interactions and reduced cross-linking, which consequently increased WVP. However, in the current study, the addition of higher concentrations of thymol (7 and 10 wt.%) resulted in a reduced WVP. This can be attributed to the overall property improvements of the films by the modified preparation method as well as by the new formulations, especially in improving the blending of thymol as a hydrophobic lipid component into the hydrophilic starch matrix. The thymol was homogeneously dispersed in the film matrix favored the cross-linking and intermolecular interactions within the starch network, thus increasing the hydrophobicity of the films and hence decreasing the WVP.

In addition, Abdollahi et al. [35] also reported that the WVP of carboxymethyl cellulose/agar films reduced with the incorporation of 0.5% *v/v* summer savory essential oil, but significantly increased when the concentration of summer savory essential oil was increased from 0.5% *v/v* to 1 and 1.5% *v*/*v*. In this case, the permeability depends on the films’ microstructure upon the inclusion of different concentrations of oil as well as the homogeneity of the oil in the matrix of the films. These results showed the variability in trends of the WVP of films incorporated with essential oils. Overall, the phenomenon of WVP is rather complicated because there are many factors that contribute to the efficiency of the hydrophobic compound and permeability of the films, including the chemical structure, morphology of the film, preparation method, type and concentration of additives, as well as the dispersion of the additives.

In this work, it was found that the WVP of the starch/CNF bionanocomposite films decreased significantly by about 60% when 7 and 10 wt.% thymol was added into the films. This result seems to be consistent with the work of López-Mata et al. [36], who incorporated carvacrol into chitosan films, whereby the WVP of the films decreased with the increment of carvacrol content. This can be explained by the hydrophobic nature of the phenolic compounds that become dominant. In their research, Lee et al. [34] revealed an increasing trend of the water contact angle of an edible film made from mung bean starch and incorporated with sunflower seed oil (SSO) at 0.5 and 1% *w*/*w*, indicating increasing surface hydrophobicity. However, the hydrophobicity was compromised at 2% *w*/*w* of SSO due to the migration of glycerol from inside to the film surface, forming a super-hydrophilic state and abruptly reducing the water contact angle. An increase in the water contact angle with the incorporation of essential oils in starch-based films was also observed by other researchers [35,37].

Meanwhile, a study conducted by Syafiq et al. [37] on the water absorption capacity of sugar palm starch (SPS)/nanocellulose crystal films incorporated with cinnamon essential oil (CEO) supports the findings of the current work. Syafiq et al. [37] found that the resistance of the films to water compared to the film without CEO was due to the degree of the film swelling and reduction of water absorption with the incorporation of 0.8 to 2 wt.% CEO into the SPS-based nanocomposite films. The combination of nanomaterial and CEO was able to stabilize and control the moisture absorption, which is able to reduce WVP.

Nonetheless, from the findings in this work, it seems like there is a certain range of concentration that resulted in the reduction of WVP. For certain types of food, such as dry food (cereal, bread, etc.), it is favorable to reduce the WVP of food packaging material, as the food packaging material should not facilitate water transfer between the food product and the surroundings, in order to enhance the shelf-life of food products.

### 3.4. Antibacterial Properties

**Figure 5:**
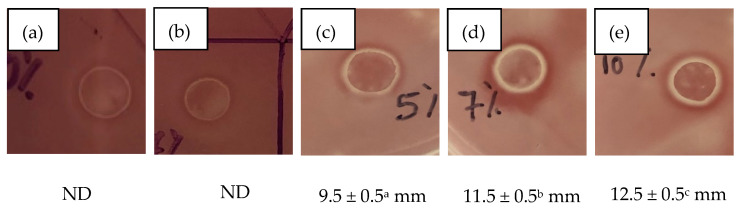
Antimicrobial activity of starch/CNF bionanocomposite films after 24 h of incubation at 37 °C incorporated with: (**a**) 0, (**b**) 3, (**c**) 5, (**d**) 7, and (**e**) 10 wt.% thymol. ND: No inhibition zone was detected. Different superscripts indicate a statistically significant difference (*p* < 0.05).

The antibacterial properties of the starch/CNF bionanocomposite films incorporated with different concentrations of thymol were demonstrated in vitro through the disc diffusion method against *E. coli* bacteria. The images of the inhibition zones that correspond to a halo around the films, including the measured diameters of the clear zone of inhibition and the films, are shown in Figure 5.

Figure 5 demonstrated that the area with direct contact with starch/CNF bionanocomposite films without thymol addition (a) and with the addition of 3 wt.% thymol (b) exhibited no inhibition zone of *E. coli*. Nonetheless, the addition of 5 wt.% thymol into the film resulting in 9.5 mm clear inhibition around the film. The diameter of the inhibition zone against *E. coli* became more pronounced with the increase in thymol concentrations. The starch/CNF bionanocomposite film incorporated with 10 wt.% thymol exhibited the strongest antibacterial effect, as demonstrated by the largest clear zone of inhibition around the film (12.5 mm). These results were consistent with the findings of Tawakkal et al. [16], who found that when the concentration of thymol was increased from 10% to 30% w/w in the PLA/kenaf films, the diameter of the inhibition zone increased from 7.5 mm to 20.6 mm at 20% *w*/*w* kenaf content. This was attributed to the volatile nature of thymol [22]. The release of the thymol, which is an antimicrobial agent, from the films to the agar medium inhibits the growth of *E. coli*. Meanwhile, the film incorporated with 3 wt.% thymol might not exhibit the inhibition zone due to the low concentration of thymol, which was not enough to inhibit the growth of *E. coli*.

These findings were consistent with the work of Petchwattana and Naknaen [25], where they found that the minimum concentration of thymol in the gelatin films that resulted in the inhibition zone of *E. coli* was 10 wt.%. However, when tested against *Staphylococcus aureus* (gram-positive bacteria), the antimicrobial activity of the gelatin films was evidenced at 6 wt.% of thymol concentration. According to Zhong et al. [22], gram-negative bacteria are less sensitive to essential oils and their components compared to gram-positive bacteria. The cell membrane of the gram-negative bacteria is indeed stronger than that of gram-positive bacteria [24]. Therefore, a higher amount of antimicrobial agents is required to tackle this limitation.

**Figure 6:**
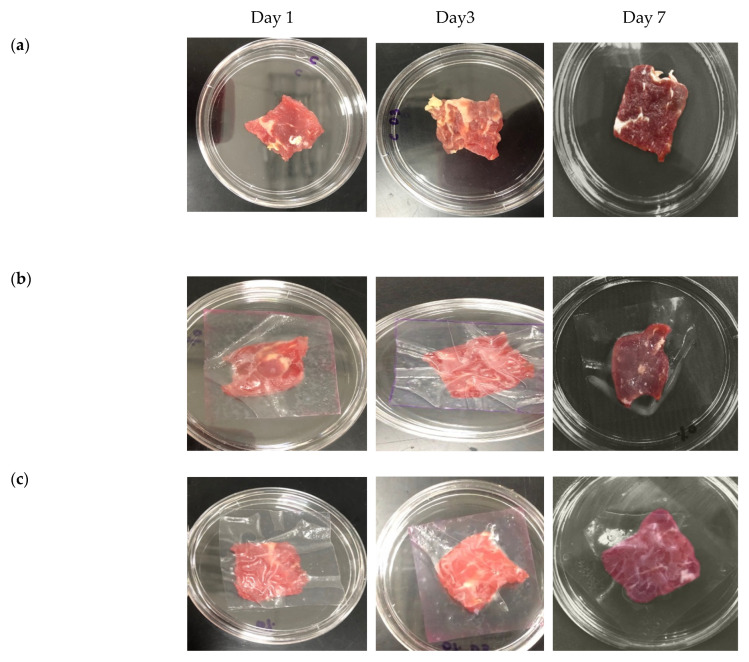
Physical appearance of the meat in direct contact with the films, stored at 13 °C on days 1, 3, and 7 (**a**) control (no films), (**b**) starch/CNF, (**c**) starch/CNF/thymol-10 wt.%.

To demonstrate the applicability of the films as potential active food packaging materials, the application of starch/CNF bionanocomposite films without and with the addition of 10 wt.% thymol was demonstrated on fresh meat samples (in vivo). The physical appearance of the meat upon storage is presented in Figure 6. As shown in Figure 6, the physical appearance of the meat changed over time depending on whether or not the meat was in direct contact with the films, as well as the addition of thymol into the film. It was clear that on day 1, all of the meat samples were visually reddish, attesting to their freshness. However, on day 3, the control meat sample started to become a bit darker in color, indicating a slight deterioration in the freshness. On day 7, the color of the meat sample became even darker, compared to day 3, demonstrating a high reduction in the freshness. The meat sample that was in direct contact with the starch/CNF film without thymol also started to present a change in its freshness as can be observed from the change in color on day 3, and the change became more pronounced on day 7. On the other hand, the meat sample that was in direct contact with the starch/CNF film incorporated with 10 wt.% thymol looked fresher and reddish on day 0 and day 3. However, the color started to change to become darker on day 7, indicating the reduction in the freshness of the meat sample.

The shelf-life of the meat sample that was in direct contact with starch/CNF film incorporated with 10 wt.% thymol was extended due to the presence of thymol as an antimicrobial agent, which slowed down the growth of bacteria that can spoil the meat. This phenomenon can also be correlated to the lower WVP of the films containing thymol, compared to the films without thymol. The lower WVP limits the exchange of water molecules between the products and the surroundings, thereby extending the freshness of the meat. It is worth noting that thymol also works as an antioxidant, and is able to minimize the change of color of the meat due to oxidation, thus leading to less color change of the meat in direct contact with the film incorporated with 10 wt.% thymol.

The efficacy of the film to preserve the meat can also be correlated to the film’s barrier to oxygen by the presence of CNF and thymol. This may be attributed to the appropriate inclusion of CNF/thymol emulsion, which formed good compatibility with the film matrix, and led to the development of a compact and dense structure, which can make it more difficult for non-polar oxygen molecules to enter the film mixture [38]. A previous study by Sun et al. [38] reported that owing to the addition of CEO at 10% to 30% *w*/*w* to sodium starch octenyl succinate films, the oxygen permeability (OP) of the films reduced from 1.61 to 1.24 10^−5^ (cm^3^·m^−2^·d^−1^·Pa^−1^). However, at higher concentrations (>30% *w*/*w*), the OP values increased due to the solubility of oxygen as a nonpolar compound in the hydrophobic EO, compromising the balance of hydrophobic/hydrophilic ratio. In addition, antioxidants with adequate free radical scavenging activity can have a positive effect on the oxygen barrier properties of films [39].

Table 2 tabulates the population of *E. coli* in terms of log CFU/m^2^ on the inoculated meat samples stored at 13 °C on three different reading days (days 1, 3, and 7). The results in Table 2 demonstrate that the antibacterial activity was the highest for the film with the addition of 10 wt.% thymol, whereby the *E. coli* population was the lowest for that film compared to the control and the film without the addition of thymol throughout the 7 days of storage. It was obvious that from day 3, the population of *E. coli* was higher for the control (fresh meat sample without film) and for the meat sample that was in direct contact with the starch/CNF bionanocomposite film without the addition of thymol, compared to that of the starch/CNF/thymol-10 wt.%.

The population of *E. coli* for the control meat sample decreased slightly from day 3 to day 7. This was most probably due to the low-temperature environment (13 °C) that was not optimal for the growth of *E. coli*. A mild temperature like 37 °C is more favorable for the metabolism of *E. coli*. However, *E. coli* has the ability to adapt and survive at low temperatures [40], hence the slight decrement in the *E. coli* population. Meanwhile, the population of *E. coli* in the meat sample that was in direct contact with starch/CNF film without the addition of thymol was slightly lower than the control due to the existence of the film that acted as a barrier against the meat sample and the surroundings. On the other hand, the population of *E. coli* decreased significantly for the meat sample that was in direct contact with the starch/CNF/thymol-10 wt.% bionanocomposite film, whereby on day 7, the population was 2.58 log CFU/cm^2^ of *E. coli*, due to the antimicrobial properties of thymol in the film.

These results were consistent with the findings of Zhang et al. [41], whereby thyme essential oil was blended in curdlan/polyvinyl alcohol composites and the resulting films were tested on chilled meat. They observed that the microbial growth was inhibited and the storage period was extended with the incorporation of thyme essential oil into the films. The bacterial count of the control group, on the other hand, increased with the storage time, and the meat was deteriorated by day 6. Furthermore, in a study conducted by Tawakkal et al. [16], it was found that the population of *E. coli* decreased significantly upon the increase in thymol concentration from 10% to 30% *w*/*w* in PLA/kenaf films. For instance, by day 19 of reading, there were no colonies detected for the samples wrapped in the films containing 20% *w*/*w* kenaf and 30% *w*/*w* thymol. This was due to the death of the bacteria caused by the destruction of their phospholipid membrane. The antibacterial activity of the starch/CNF/thymol bionanocomposite films via in-vitro and in vivo (*E. coli* counts) analyses proved the potential of the films to increase the shelf-life of the meat sample.

## 4. Conclusions

Starch/CNF bionanocomposite films incorporated with different concentrations of thymol were successfully produced and investigated. The addition of thymol slightly increased the thickness, ΔE values, and opacity of the films. In terms of the mechanical properties, TS and YM decreased while EAB increased with the increase in thymol concentrations in the starch/CNF/thymol bionanocomposite films, indicating the improvement of the flexibility and the decrease in the rigidity due to the plasticizing effect of thymol. It was found that the mechanical properties of the starch/CNF/thymol bionanocomposite films produced in this study were in accordance with that of some common food plastic materials, proving that they are suitable, promising, and exhibit high potential for food packaging applications, particularly as flexible packaging. The incorporation of thymol at high concentrations (≥7 wt.%) into the starch/CNF bionanocomposite films led to a significant decrease in the WVP, due to the hydrophobic nature of the phenolic compounds in thymol. Meanwhile, the inhibition zone against *E. coli* was observed when the films were added with 5 wt.% and higher concentrations of thymol. The diameter of the inhibition zone increased with the increase in thymol concentration. The in vivo analysis demonstrated that the starch/CNF/thymol-10 wt.% bionanocomposite film inhibited microbial growth and reduced the bacterial count on the fresh meat samples. The produced films exhibit the potential to be used as active food packaging materials to enhance the shelf-life of beef and chicken meat slices. Future investigations can be aimed at examining the thermal properties and biodegradability of the films, which are particularly important for applications. The overall properties of the films can also be improved by devising the film formulation with other functional ingredients, such as antioxidant agents, vitamins, or enzymes. It is also recommended to demonstrate the application of the starch/CNF films incorporated with thymol as antimicrobial packaging to a larger scale and investigate the antimicrobial activity of the films against different types of microorganisms that are relevant to food.

## Figures and Tables

**Table 1:** Thickness, color parameters, and opacity of starch/CNF bionanocomposite films incorporated with different concentrations of thymol.

Concentration of Thymol (wt.%)	Thickness (mm)	L*	a*	b*	ΔE	Opacity (A.mm^−1^)
0	0.0859 ± 0.0004 ^a^	92.11 ± 0.26 ^a^	−0.93 ± 0.01 ^a^	5.46 ± 0.19 ^a^	-	8.824 ± 0.63 ^a^
3	0.0882 ± 0.0010 ^b^	93.02 ± 0.34 ^bc^	−0.89 ± 0.01 ^b^	4.25 ± 0.64 ^b^	1.514 ± 0.06 ^a^	8.891 ± 0.64 ^a^
5	0.0907 ± 0.0010 ^c^	93.53 ± 0.27 ^c^	−0.88 ± 0.01 ^b^	4.15 ± 0.75 ^b^	1.933 ± 0.10 ^b^	9.209 ± 0.40 ^ab^
7	0.0925 ± 0.0006 ^d^	93.61 ± 0.51 ^c^	−0.95 ± 0.02 ^ac^	4.07 ± 0.87 ^b^	2.045 ± 0.11 ^bc^	9.410 ± 0.39 ^ab^
10	0.0945 ± 0.0008 ^e^	93.68 ± 0.76 ^c^	−0.95 ± 0.01 ^c^	4.12 ± 0.25 ^b^	2.064 ± 0.04 ^c^	9.678 ± 0.65 ^b^

Different letters in the same column of the table indicate a statistically significant difference (*p* < 0.05).

**Table 2:** Antibacterial activity of control, starch/CNF bionanocomposite film, and starch/CNF/thymol bionanocomposite film against *E. coli* on fresh meat samples.

*E. coli* Counts (log CFU/cm^2^)			
Films	Day 3	Day 5	Day 7
Control (no film)	9.52 ^Aa^	9.51 ^Aa^	8.34 ^Ab^
Starch/CNF	9.31 ^ABa^	9.29 ^ABa^	8.32 ^Ab^
Starch/CNF/thymol-10 wt.%	8.13 ^Ca^	6.12 ^Cb^	2.58 ^Bc^

Different letters in the same column and row of the table indicate a statistically significant difference (*p* < 0.05).
